# Developing a Smart Home Technology Innovation for People With Physical and Mental Health Problems: Considerations and Recommendations

**DOI:** 10.2196/25116

**Published:** 2022-04-29

**Authors:** Cheryl Forchuk, Jonathan Serrato, Daniel Lizotte, Rupinder Mann, Gavin Taylor, Sara Husni

**Affiliations:** 1 Mental Health Nursing Research Alliance Parkwood Institute Lawson Health Research Institute London, ON Canada; 2 Arthur Labatt Family School of Nursing Western University London, ON Canada; 3 Department of Computer Science Western University London, ON Canada; 4 Research Informatics Department Lawson Health Research Institute London, ON Canada; 5 Information Technology Services London Health Sciences Centre London, ON Canada

**Keywords:** smart home, smart technology, mental health, physical health, eHealth, comorbidity, innovation, communication, connection, uHealth, ubiquitous health, digital health

## Abstract

Smart home technologies present an unprecedented opportunity to improve health and health care by providing greater communication and connectivity with services and care providers and by supporting the daily activities of people managing both mental and physical health problems. Based on our experience from conducting smart technology health studies, including a smart home intervention, we provide guidance on developing and implementing such interventions. First, we describe the need for an overarching principle of security and privacy that must be attended to in all aspects of such a project. We then describe 4 key steps in developing a successful smart home innovation for people with mental and physical health conditions. These include (1) setting up the digital infrastructure, (2) ensuring the components of the system communicate, (3) ensuring that the system is designed for the intended population, and (4) engaging stakeholders. Recommendations on how to approach each of these steps are provided along with suggested literature that addresses additional considerations, guidelines, and equipment selection in more depth.

## Introduction

Smart home technologies present an unprecedented opportunity to improve health and health care. Enabled by the accessibility of wireless networks and computing devices inserted into everyday objects to create the internet of things [1], smart home technologies allow health practitioners to capture and monitor real-time data where behavioral anomalies such as irregular sleep patterns, inactivity, high heart rate levels, and sudden increase/decrease of weight could be indicative of psychological distress or the early stages of a health crisis. Within vulnerable populations, instrumental activities of daily living such as handling medication and self-care routines can start to decline rapidly [2,3]. By enabling health care personnel to remotely monitor physiological and mood changes in real time and by supporting communication between patients and providers, smart home technologies can mitigate this decline by allowing individuals to manage their symptoms at home instead of waiting to visit health care facilities.

Health care systems are strained by the demand for mental and physical health care. Despite unprecedented federal investment in mental health and addiction services [4], over 2.3 million Canadians report unmet or partially met health care needs [5]. Demand for physical and mental health care has been exacerbated by the COVID-19 pandemic, even as it has increased barriers to access [6,7]. Smart home technologies can potentially reduce this demand by enabling users to monitor their health and live healthier lifestyles [8], particularly because physical activity has significantly declined in response to public health restrictions [9]. Reframing the health care system to deliver better coordinated, accessible care requires not only increased funds but also embracing innovative models of care delivery such as those afforded by smart home systems.

Beyond direct benefits to patients and the health care system, smart homes offer potentially significant wider economic benefits. While there may be upfront costs in purchasing, developing, and integrating the technology, there is potential for the innovation to be cost effective in the long term through a reduction in usage and resources for services. Researchers have reported a 38% decrease in administration costs pertaining to billing for care provisioning and care rescheduling within 8 years of introducing smart home care platforms [10]. There is the potential that smart technologies in the home would be helpful for individuals who require intensive support and experience barriers to, or disparities in, community care such as geographical location and inadequate travel arrangements, discrimination and stigma, and lack of resources [11].

Although technological studies investigating the use of apps [12], smartphones [13], and robotics [14] for mental health care exist, as well as for physical conditions such as Parkinson disease [15] and diabetes [16], research that incorporates aspects of both mental and physical health in one study remains limited, as is guidance for developing and implementing smart home–based interventions intended to support both mental and physical health. The contribution of this paper is to provide recommendations and considerations for future research projects looking to develop a smart technology innovation for mental and physical health care.

**Figure 1:**
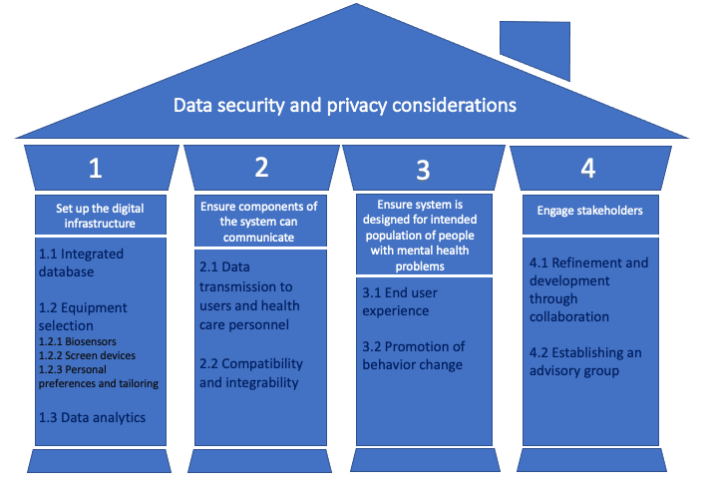
Steps and considerations for a smart home technology innovation.

Reflecting on our own experience in this area [17], we have identified 4 key steps for developing a successful smart home–based intervention for supporting mental and physical health and health care that are outlined in Figure 1: (1) set up the digital infrastructure; (2) ensure components of the system can communicate; (3) ensure that the system is designed for the intended population; and (4) engage stakeholders. These 4 key steps are embedded within the overarching principle of data security and privacy. In the following, we describe each of the steps in detail and how they fit within this overarching principle, with the goal of supporting future research and implementation of smart home technologies for health.

## Data Security and Privacy Considerations

Crucial to any smart home innovation is the issue of data security and privacy by design. There are many challenges to maintaining anonymity and privacy. Even after data have been deidentified, there is still a risk of data linking the health record to the individual (eg, through a combination of location and rare attribute values). Recent research into data anonymized using Health Canada regulations has reported death records and social media to be the most likely sources of reidentification [18]. Furthermore, there are legal requirements in different jurisdictions that must be attended to in any project that collects data through smart devices.

In Canada, the provincial government of Ontario enacted the Personal Health Information Protection Act (PHIPA), a law that aims to maintain the security and privacy of personal health information of Ontarians by ensuring protection against unauthorized usage, theft, loss, or disclosure. Under this Act, agencies and organizations can send and receive deidentified health data, provided it is for the purposes of health system delivery, design, or planning. PHIPA is not only applicable to health information custodians, but also to custodians who have received health data, including health care providers and researchers. To ensure these standards are met, PHIPA asserts 10 fundamental principles [19]: (1) accountability; (2) identifying purposes; (3) knowledge and consent; (4) limiting collection; (5) limiting use, disclosure, and retention; (6) accuracy; (7) safeguards; (8) openness; (9) individual access; and (10) challenging compliance.

While PHIPA is specific to Ontario, there are many laws worldwide that hold similar principles [20,21]. It is important that these 10 principles are considered and followed when designing a smart technology innovation, both to ensure compliance with current governance and to enhance the efficacy, security, and privacy of data within a technology-based study. In the research environment, it is crucial that data be kept secure and participant identities remain anonymous. Further, in Ontario, the research team is required by PHIPA to fully investigate and report any form of breaches or losses of participant information to those participants identified in the data. In 2005, electronic health records require the consent of patients prior to their storage as outlined in the Personal Information Protection and Electronic Documents Act (PIPEDA) in Canada. This Act sets out to ensure that data are deidentified, and that personal information cannot be identifiable by a member of the public. Data linkages between organizations, whether they be private or public sector, are also governed by PIPEDA. However, this differs from PHIPA in that PIPEDA covers all data, whereas PHIPA focuses on health information data within Ontario. In the United States, the US Health Insurance Portability and Accountability Act (HIPAA) provides a set of guidelines for personal health data [22].

It should be highlighted that data stored on clouds, defined as a computing model that enables access to be shared or saved data regardless of the user’s location or device used [23], may be subject to government inspection or surveillance depending on where the cloud servers are located. Under the Patriot Act (also known as the Uniting and Strengthening America by Providing Appropriate Tools Required to Intercept and Obstruct Terrorism Act of 2001), US law enforcement can issue a FISA (Foreign Intelligence Surveillance Act) Order or National Security Letter to any company that has a US office, US-based server, or conducts business within the United States, even if the data under investigation are stored on a server outside of the United States [24]. Similarly, the CLOUD (Clarifying Lawful Overseas Use of Data) Act allows US law enforcement to access the private data of American citizens stored on foreign servers [25]. In China, the passing of the Cybersecurity Law in November 2016, which legislated further cyberspace governance of data stored locally on servers based within China, has also led to tension and suspicion of government interference [26]. It is therefore important for potential studies to be aware of sensitive data being inspected by government agencies. It is recommended that researchers be aware of the data they are collecting and if they wish to maintain privacy, and that equipment and services are carefully selected.

A smart home innovation can be at risk of privacy breach depending on the security of the chosen wireless connectivity, for example, Wi-Fi, 3G, 4G, LTE (Long-Term Evolution), radio frequency identification, or wireless sensor networks [27] Wireless sensors and devices can be vulnerable to network-based attacks, resulting in exposure of personal information or malfunction. End-to-end encryption of all transmitted data and health records through tried and tested security protocols such as single sockets layer (SSL) can ensure the integrity and privacy of information and device operation. Additionally, any data integration across platforms must be accomplished without compromising privacy and integrity [28].

Finally, it is worth considering that there are the interrelationships between users within a home and the privacy boundaries that can arise when sharing devices among users or when technologies are situated in a shared space. An ethnographic study reported that one way of maintaining boundaries with technology within the smart home environment is to keep some rooms “technology free” [29]; this may be a valuable approach in certain shared-use situations. Individuals should be given the opportunity to share data with caregivers and members of their care team as well as be able to maintain privacy, both virtually and within the lived environment.

Having established the overarching principles of data security and privacy by design, we will next present our 4 steps for developing a successful smart home innovation.

## Steps for Developing a Successful Smart Home Innovation

### Set Up the Digital Infrastructure

#### Integrated Database

Simply monitoring the data coming from a multitude of devices and the devices’ respective clouds or apps is not ideal for a health care professional, because it provides a disjointed view of a client’s information. A centralized, integrated database capable of funneling the various forms of real-time data into one accessible location can provide a more efficient approach to monitoring and tracking participant health data. Jensen [30] highlighted 5 key principles in designing a centralized integrated database: ease of use and implementation, built upon existing databases, expand installed base by persuasive tactics to gain momentum, making compatibility as simple as possible, and modularizing information infrastructures (ie, changes made do not affect the entire infrastructure).

Moreover, a centralized database can have other benefits such as providing a baseline rate of technology use and user activities whereby change can be observed and measured over time. From a research and a care perspective, this would provide an empirical basis for observation of mental and physical health progress/decline as well as precursors to health crises.

Security and privacy of a centralized integrated database can elicit concerns from health care providers and participants. This was evident in Iceland when 11% of the population opted out of a national database, causing the initiative to be scrapped [31]. This highlights the importance of ensuring that individual autonomy is respected and participants are well informed about the potential risks and benefits of their participation to obtain an informed consent. The consent process can be used to allay any fears of data mismanagement and provide assurances of data security. The participants need to understand how their data will be used and whether it will be for primary or secondary analyses. It is important to ensure that only authorized personnel, such as the participant’s care provider, can access the health data and that this is clearly explained to both participants and care providers.

Outside of data security, there may also be concerns of a power failure or server crash that would compromise live data or result in a lack of access for care providers. It is recommended that administrators and technicians for an integrated database consider combining environmental and network redundancy with a robust virtual server environment to enable continuous operations with minimal or no impact on usage or data transmission. This can also be utilized during peak overloads or during maintenance to ensure functionality.

#### Equipment Selection

##### Overview

In the following, we discuss the selection of biosensors and screen devices, and we discuss the issue of tailoring devices to the needs of populations. In our experience, for all of these types of devices, it is advantageous to choose screen devices and biosensors that are easily and commercially available (ie, able to be purchased in shops/online) as opposed to expensive clinical equipment that are harder to purchase or replace.

##### Biosensors—Weigh Scales, Blood Pressure Monitors, Glucometers, and Wearable Activity Trackers

The interplay of physical and mental health conditions often complicate help-seeking and adherence to treatment, which worsens the prognosis of all present diseases [32,33]. The prevalence of chronic physical conditions, such as diabetes and cardiovascular disease, are higher among people with mental illness [34,35]. To account for the rise in comorbidities, a comprehensive smart home intervention equipped with noninvasive and nonintrusive sensors (wearable activity trackers, glucometers, and weigh scales) can be viable diagnostic tools for health care professionals [36,37]. The embedded sensors within these devices capture important physiological parameters such as cardiorespiratory function, blood sugar levels, weight/BMI, gait analysis, and sleep patterns that are then funneled into a centralized database, capable of logging and analyzing the collected data for anomalies.

Wearable sensor-based health monitoring systems should achieve maximal usability and reduce operator discomfort. Sensors that are embedded into textile fabrics or surface mounted directly to the body require a stable sensor-skin interface to ensure high signal accuracy and durability [36]. From an ergonomic standpoint, the selected hardware should be comfortable, flexible, small, and unobtrusive when attached to the body. It should also ensure minimal risk of harm to users and compliant with industry standards (ie, nontoxic, nonreactive, and manufactured from hypoallergic materials).

These devices should have low power requirements and should exhibit “always-on” connectivity as frequent removal for charging or syncing prevents seamless integration with daily activity and presents a barrier to long-term utilization. Tech-enabled, noncritical monitoring used in tandem with standard health services has potential not only to alleviate the workload on health care providers, but also can be used to inform accurate symptom reports, diagnoses, and prompt referrals to specialized care.

##### Screen Devices—Smartphones, Tablets, and Monitors; Apps

Connectivity with care providers and access to resources have become increasingly enhanced with modern hardware and software. Research has reported that usage can be measured (ie, number of SMS text messages sent, physical movement) to predict mood disorder changes [38]. The use of screen devices in combination with specially designed apps can render positive outcomes. A systematic review on mental health apps used on smartphones and tablets revealed significant reductions in depression, stress, and substance use [39]. Another review of physical health and self-care apps revealed an increase in physical activity, weight management, smoking cessation, and medication adherence with favorable feasibility and usability [40]. We recommend that smart home technology interventions include screen devices, such as tablets, monitors, or smartphones. Screen devices should be equipped with secure apps to support remote telemonitoring and provide access to personal health information. They should also provide secure communication between participants and their health care providers (including electronic messaging and videoconferencing) and provide the ability to customize and display screen prompts including real-time notifications to facilitate self-care.

Consequently, health care providers can respond swiftly to significant deviations in the patient’s normal vital signs by notifying the patient through the screen devices and scheduling ad hoc virtual care. Communication through the screen devices should be bidirectional so patients can not only acknowledge and respond to notifications from their health care providers, but can also request additional help or support, if required.

Screen devices should also include features that support mood tracking, such as standard or client-specific self-assessments. Not only does mood tracking reduce the logistic burden of data collection, but it also has the potential to enrich clinical practice by offering novel methods of monitoring psychopathology [41]. Conventional, cross-sectional surveys and clinical interactions have limited impact because they collect retrospective rather than real-time data. Mobile apps with mood-tracking features represent a feasible method for capturing “in the moment” patient data through experience sampling methods [42]. Changes in mood could also be reflective of other physical health symptomology that may be present or alleviated. This provides a more detailed understanding of mental health phenomenology because daily fluctuations in mood are linked to time-stamped activities and social contexts, thereby elucidating the dynamic relationship between environment and symptomatic experiences [41-43]. A technological innovation using mood tracking technology in combination with biosensors can further outline the bidirectional effects of mental and physical health.

##### Consideration of Personal Preferences and Tailoring

Models of intervention delivery leveraging the ubiquity of technological access must be tailored to the unique challenges faced by people with mental and physical health problems in order to be successful. To be most impactful, components of the intervention should be client tailored and thus, developed and integrated under a framework of validated health behavior theories [44]. In the Lawson Health Smart Home study [17], participants were able to add/remove equipment as and when was needed, or due to personal preference. For example, one participant originally declined the medication dispenser but then later requested it to assist with medication adherence. The dispenser was installed and the participant reported no missed doses thereafter.

It is very important to consider the potential negative consequences on mental health following the introduction of technology. For example, the use of smart mirrors (mirrors with touchscreen and connectivity capabilities) and voice-activated devices could exacerbate paranoia or delusions. Although user preference is paramount, clinical decision makers are needed for input into the appropriateness of devices. The introduction of technology should provide support and care, and smart technology interventions must be flexible in accounting for specific illnesses, symptoms, and comorbidities.

#### Data Analytics

Health apps can provide analytical data to users by monitoring their health and providing feedback. Dimitrov [45] noted that the use of artificial intelligence aims to identify and analyze patterns of data from one individual and then compare those with similar patterns to make predictive recommendations, thereby acting as a personal coach to the individual. An example of this would be number of steps or calories burned, and how that aligns with the individual’s goals and targets. It could be further argued that appropriate application of artificial intelligence would help triage patients by empowering participants and lessening the burden on health care services and clinicians [45]. Data derived from health devices can be combined with existing data sets to provide greater contextual understandings. Although analytic capabilities are useful, it is crucial to remember that population and individual metrics can vary differently and can be determined through differing methodologies/models. For example, blood pressure monitoring would not be generalizable to the population due to measurement errors based on the time of day [46]. Taking individual factors into account would be a key recommendation when developing an algorithm or a predictive model as errors can occur and erroneous assumptions could be made.

It is recommended that future research studies follow these 6 best practice steps [47] for analyzing health data from wearable devices and health apps:

1. set a robust research question where access to the necessary data is available, including the data on the device and publicly available data sets for comparisons (eg, life expectancy, prevalence rates);
2. prepare data for analysis to anticipate inaccuracies and missing data as well as removing outliers and erroneous values, ascertaining sensor accuracy, and performing trial runs prior to implementation;
3. verify the data set by comparing with current literature and gold-standard data sets to ensure consistency or find inconsistencies;
4. analyze data that answer the research question(s), establishing causal relationships and the possibility of needing propensity scoring models to minimize confounding variables;
5. check the robustness of conclusions and establish the validity of the findings;
6. knowledge dissemination including sharing data sets and coding while maintaining privacy and governance standards.

In the Lawson Health Smart Home innovation, questionnaires were programmed so that if a participant expressed suicidal thoughts or ideations, a notification was immediately sent by SMS text message or email to their care provider(s). It is important for any smart technology study that trends can be observed and saved for future analyses and to provide a bigger picture of the participant’s current health with contingencies in the event there are concerning data trends.

### Ensure Components of the System Can Communicate

#### Data Transmission to Users and Health Care Personnel

Unprocessed data captured by noninvasive sensors are transmitted to a processing node using a low-power and short-range communication protocol such as Bluetooth, ZigBee, ANT, or near-field communications (NFC) [36,48-53]. The processing node is an advanced platform that first collects and filters the data, and then executes advanced analysis and decision algorithms. Common examples include smartphones, tablets, computers, and personal digital assistants. The processing node functions as a gateway, or a central hub, that receives raw data input from wearables and transmits measured data output to a secure server located in a remote health care facility. Depending on what processing node is used, results may be stored, translated, and displayed on a user interface. Interventions consisting of only a few sensors can send data directly to the processing node, while more complex systems with several sensor units can first gather data through a central body area network [36]. Processed data transmitted to the hospital server over the internet requires a long-range communication protocol.

To ensure data transmission, the processing node should achieve reliable, stable data transmission without any interference and present a low risk of being hacked. In a multisensor body area network system, the central processing node requires a significant amount of power to handle the large influx of data; therefore, power consumption levels must be lowered to sustain long-term utilization [36]. Likewise, the transmission of measured data outputted from the processing node to a centralized integrated database through the internet will require appropriate encryption (ie, public key infrastructure, SSL), authorization, and authentication algorithms to ensure personal health information is secure and protected [36,54,55].

#### Compatibility and Integrability

**Figure 2:**
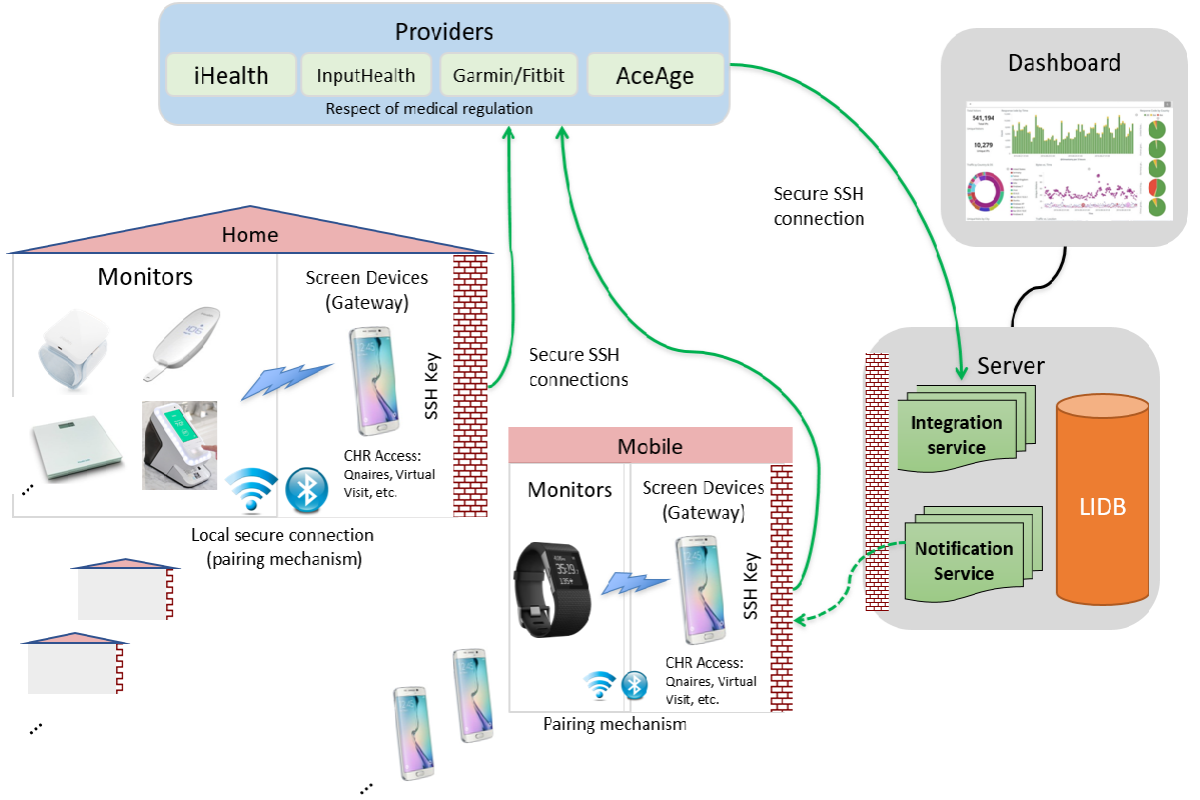
System architecture for the Lawson Smart Homes Innovation [17]. CHR: collaborative health record; LIDB: Lawson Integrated Database; Qnaires: Questionnaires from CHR; SSH: secure shell.

Compatibility is a key issue when connecting multiple devices for a smart technology innovation. A major aspect of building a smart technology innovation is for the individual pieces of equipment to be able to communicate with one another, or that they are all compatible with 1 central data center. See Figure 2 for an example of the smart home system from the Lawson Health study [17]. With multiple operating systems currently available (eg, Android, iOS, Windows, Linux, Raspberry), it is crucial that the technologies are capable of interacting with one another and able to transmit data seamlessly without any drop in performance. To implement a centralized integration of data originating from different sources, it is essential to consider software formats so that the end users can be provided with a translated and unified view of the accumulated data. It may require the data extraction and synchronization processes, considering data might be coming from myriad sources using different transmission schedules and rates. This is where implementation of processes such as ETL (extract, transform, load) is advocated. Integrability is a key component of developing a smart home innovation as individual devices need to be integrated seamlessly into the individual’s care plan as well as meeting the purposes of the system. Furthermore, a smart home innovation could result in the integration of multiple members of the care team or additional service providers consulting on all of the data collected as opposed to their own individualized data.

### Ensure That the System Is Designed for the Intended Population

#### End User Experience

Smart technology interventions for people with mental and physical health problems should be designed with the goal of optimizing the user experience. It is imperative that interactions with the selected equipment are as simple and efficient as possible. A smart home innovation should connect devices to anticipate user needs, automate complex configurations, and be “smart” enough to make independent, intelligent decisions. The limited space for micro interactions on wearable devices should be intuitively optimized so that features can be easily navigated on the main menu [56]. In addition, the innovation should have a micro feedback display that provides immediate, quantitative feedback on biometrics related to the users’ exercise quality, cardiorespiratory function, and sleep patterns.

On the hub, the data are processed and translated into usable information and the results are displayed on a mobile app that synchronizes to the wearable hardware. The mobile app is the component with which users most frequently interact and should connect with the entire suite of selected wearable devices. The app should be able to chart their progress with data visualization tools such as calendars and graphs and provide relevant information (eg, steps, distance, weight, sleep cycle) that is easily accessible and interpreted. The app interface should use colors, animations, and typography congruent with the intended purpose of a mobile app and embrace minimal cognitive overload principles (ie, avoid too much content, irrelevant steps, or inconsistent formatting).

#### Promotion of Behavior Change

In addition to presenting the data, devices should include functionality that promotes sustained behavior change where appropriate. Based on principles of positive reinforcement, strategies to engage users should address all 3 components of the habit formation loop [56]: cues (push notifications, alarms, vibrations), behaviors (prompts to exercise, sleep, hydrate), and rewards (badges, level progressions, gamification). Research has shown that sharing personal information with friends and family increases the likelihood of attaining goals [57,58]. Mobile apps selected or developed for smart home interventions should support additional aspects of care provisions such as secure virtual care (messaging, audio, and visual), mood tracking features, and active prompts for medication, activity, or appointment reminders.

### Engage Stakeholders

#### Refinement and Development Through Collaboration

Consultation with stakeholders is a crucial component of developing a smart technology innovation. It is important to identify from the outset as to who would have an interest in this innovation and whether they can assist in its development, refinement, and implementation. This is particularly of great value when the new innovation is untested, unproven, or may contain risks such as privacy. Hart and Sharma [59] note that many companies tend to focus on stakeholders that are known to them or hold powerful positions but fail to engage with peripheral stakeholders. This then leads to a lack of competitive imagination and positive disruptive change. It was suggested by Wright [60] that engaging stakeholders prior to deployment of new information technology can help to mitigate ethical implications by ensuring measures are taken and risks adequately examined. This is also true of research: by embracing and encouraging ideas from stakeholders outside of the research environment, the project can develop a greater depth of ideas and imagination. Roles in provision, oversight, and management of the intervention should involve an interdisciplinary team of health care providers and experts of the mental and physical illnesses the intervention is seeking to address and support.

#### Establishing an Advisory Group

It is recommended that stakeholders be encouraged to provide input throughout the design process through the formation of an advisory group. A co-design approach through the collaborative efforts of an advisory group can allow for reductions in oversights, greater suggestions for improvement, and ensuring targets are met. The objectives of an advisory group are to ensure the project remains on track, help the project overcome obstacles that may arise, and help align the technology with present and future opportunities for scaling up or further development.

**Figure 3:**
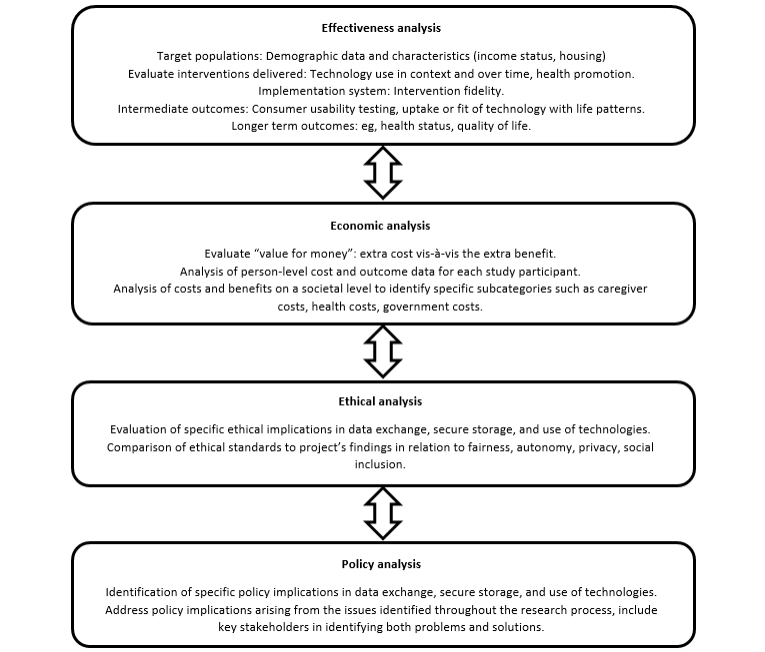
Standardized evaluation framework for smart technology mental health interventions [62].

This group ideally consists of end users, consumers, service providers, policy decision makers, industry representatives, analysts, and researchers. End users and consumers should be consulted throughout development which can involve “citizen juries” [61], focus groups, and pilot testing. As well, analysts and researchers within the advisory group should contribute to the evaluation of the intervention. The evaluation depends on the research questions and hypotheses of the project. A standardized evaluation framework for smart technology intervention that covers a broad range of implications would be recommended [62] (Figure 3). It is important that not just the effectiveness of the intervention is measured, but also inferences for sustainability beyond the study; cost is compared with usual care; and ethical considerations are obtained. In summary, a comprehensive approach to evaluation and refinement of a smart home intervention from all interested parties allows for risk management, improved quality control, and enhanced innovation.

## Conclusion

The overarching principle of data security and privacy together with our 4 steps were identified from our experience developing a smart home intervention for a target population that included end users who were particularly struggling with physical and mental health conditions. However, they are equally applicable to other interventions that address many different mental and physical health concerns. Going forward, we see huge potential for smart home research and implementation work that takes a holistic view of the end user. Smart home technologies have the potential to support all aspects of health by targeting not only health care issues but also by facilitating more social inclusion and better health behaviors, leading to improved quality of life.

## Abbreviations

CLOUD: Clarifying Lawful Overseas Use of Data Act
ETL: extract, transform, load
FISA: Foreign Intelligence Surveillance Act
HIPAA: US Health Insurance Portability and Accountability Act
LTE: Long-Term Evolution
NFC: near field communications
PHIPA: Personal Health Information Protection Act
PIPEDA: Personal Information Protection and Electronic Documents Act
SSL: single sockets layer
